# Population status, distribution and trophic implications of *Pinna nobilis* along the South-eastern Italian coast

**DOI:** 10.1038/s44185-022-00002-2

**Published:** 2022-11-17

**Authors:** Davide Pensa, Alessandra Fianchini, Luca Grosso, Daniele Ventura, Stefano Cataudella, Michele Scardi, Arnold Rakaj

**Affiliations:** 1 Experimental Ecology and Aquaculture Laboratory, Department of Biology, University of Rome Tor Vergata, Via Cracovia 1, 00133 Rome, Italy; 2 PhD Program in Evolutionary Biology and Ecology, Department of Biology, University of Rome Tor Vergata, Rome, Italy; 3 Department of Environmental Biology, University of Rome “La Sapienza”, Viale dell’Università 32, 00185 Rome, Italy; 4 National Inter-University Consortium for Marine Sciences-CoNISMa, Rome, Italy

**Keywords:** Ecology, Environmental sciences

## Abstract

The dramatic Mass Mortality Event, MME, of *Pinna nobilis* populations initially detected in the western Mediterranean basin, has also spread rapidly to the central and eastern basin. Unfortunately, there is still a significant lack of information on the status and health of *P. nobilis*, since only a fragmentary picture of the mortality rate affecting these populations is available. Regarding the Italian coast, several surveys have given only localized or point-like views on the distribution of species and the effect of the MME. Therefore, for the first time, this study investigated *P. nobilis* density of individuals, distribution and mortality throughout 161 surveys along 800 km of coastline in the Apulia region (South-east of Italy). The geographical scale of this investigation made it the largest ever conducted in Italy, and this was achieved through a rapid and standardized protocol. During this monitoring campaign, 90 km of linear underwater transects were surveyed, along which no live individuals were observed. This result allowed to estimate that the *P. nobilis* populations had totally collapsed, with a mortality rate of 100% in Apulia. The distributional pattern of the species showed a strong overlap with seagrass meadows on meso- and macro-geographical scale, however this was not the case on a micro-scale. This result evidenced that relationships between *P. nobilis* and seagrass meadows are not limited to the habitat patch, but cross the boundaries of seagrass leading us to suggest that the distribution of *P. nobilis* hold a trophic link through the cross-boundary subsidy occurring from seagrass meadows to the nearby habitat, by means of the refractory detrital pathway.

## Introduction

*Pinna nobilis* Linneaus,1758, is an endemic species of the Mediterranean Sea and is one of the largest bivalve mollusks worldwide^1^. This species filters large quantities of seawater^1,2^, retaining phytoplankton, zooplankton, and indeterminate debris^3^, but its ecological role in the benthic food chain dynamics still remains poorly understood despite the considerable densities and biomass reached in some coastal habitats^4^.

In the last 5 years, the pen shell has been disappearing due to a Mass Mortality Event (MME). The causes are still under investigation, although the protozoan, *Haplosporidium pinnae*, has been indicated as the probable main driver of the MME^5–7^. In 2016, the MME was first detected across a wide geographical area of the Spanish coast^7^, affecting specimens of all sizes in a wide range of depth and habitat type^5,7^. The disease spread rapidly from the west to east of the Mediterranean basin, reaching the coasts of France, Italy, Croatia, Greece, Turkey, and northern Africa^8,9^, causing high mortality rates in infected populations. As a consequence, in December 2019, *P. nobilis* entered the IUCN Red List as a critically endangered species^9^.

In early 2017, the MME of *P. nobilis* was reported along the western coast of Italy (Campania and Sicily), affecting 85–100% of the population^10^. In the summer of 2018, an infected population was recorded in the Mar Piccolo of Taranto^11^, and in 2019 the disease reached the coasts of the Northern Adriatic^12^. Recently, abnormal mortalities have also been reported inside the Venice lagoon, which was thought to host one of the last healthy populations of the north Adriatic Sea^13^. We currently have only fragmented knowledge of the mortality rate affecting populations, based on localized observations focused on specific areas. This is the case for the entire Mediterranean basin, except for a few studies covering large geographical areas such as Šarić et al.^14^ for the Croatian coast, Vázquez-Luis et al.^7^ and García-March et al.^15^ for the Spanish coast and Zotou et al.^16^ for the Greek coasts.

Regarding the Italian coasts, in particular, there is a lack of surveys aiming to assess the species distribution and the effect of the MME on a large geographical scale. This constitutes the most significant information gap on the state of health of *P. nobilis* populations. It is essential to gather data from large-scale monitoring activities to understand the state of local populations, the pressures, and the trends affecting them. For this reason, gaining an overview of the distribution of natural populations before the MME and on the incidence of mortality in local populations is the starting point for undertaking critical protection and management actions.

The aim of this study was to track, by means of comprehensive surveys, the progress of the MME and to provide updated information on the status of *P. nobilis* in the Apulia region (Southeastern Italy), along 800 km of both Adriatic and Ionian coastline. Furthermore, a transversal investigation on species distribution patterns in relation to the substrate type was carried out among different habitat types and ecosystems to investigate the main drivers underlying its spatial distribution. For this purpose, a rapid and standardized protocol was developed through visual census surveys.

## Results

A total of 315 recently dead specimens were recorded in 161 transects from the 30 coastal areas investigated. No live individuals were observed.

According to the three size classes reported in Table 1 (large: >60 cm; medium: between 60 and 40 cm; small: <40 cm), dead specimens of all sizes and ages (both juveniles and adults) were found at all depths and habitat types examined. No difference emerged between transitional, coastal, and island ecosystems, all submitted to total mortality.

**Table 1:** Exploited areas, habitat type, area covered, specimen densities, specimen size, and mortality rate.

Site	Locality	Basin	Environment type	Substrate type	Exploited area (m^2^)	No. of *P. nobilis*	Size (large; medium; small)	Population density (/100 m^2^)	Mortality incidence
A1	Tremiti (MPA)	Adr	Islands	*P. oceanica*, sandy, rocky	1900 m^2^	6	3 L; 2 M; 1 S	0.31	100%
A2	Lesina	Adr	Coastal	Sandy	1800 m^2^	0	0 L; 0 M; 0 S	0	n.a.
A3	Capoiale	Adr	Coastal	Sandy	1800 m^2^	0	0 L; 0 M; 0 S	0	n.a.
A4	Vieste	Adr	Coastal	Sandy, rocky	1800 m^2^	0	0 L; 0 M; 0 S	0	n.a.
A5	Manfredonia	Adr	Coastal	*C. nodosa*, sandy	1800 m^2^	0	0 L; 0 M; 0 S	0	n.a.
A6	Margherita di Savoia	Adr	Coastal	Sandy	1800 m^2^	0	0 L; 0 M; 0 S	0	n.a.
A7	Bisceglie	Adr	Coastal	*P. oceanica*, sandy, rocky	3600 m^2^	2	0 L; 1 M; 1 S	0.06	100%
A8	Mola di Bari	Adr	Coastal	*P. oceanica*, sandy, rocky	3600 m^2^	2	0 L; 0 M; 2 S	0.06	100%
A9	Monopoli	Adr	Coastal	*P. oceanica*, sandy, rocky	3000 m^2^	2	0 L; 0 M; 2 S	0.07	100%
A10	Torre Canne	Adr	Coastal	*P. oceanica*, sandy, rocky	3300 m^2^	1	0 L; 0 M; 1 S	0.03	100%
A11	Torre Guaceto (MPA)	Adr	Coastal	*P. oceanica*, sandy, rocky	3100 m^2^	4	0 L; 2 M; 2 S	0.13	100%
A12	Brindisi	Adr	Coastal	*P. oceanica*, sandy, rocky	5300 m^2^	2	0 L; 2 M; 0 S	0.04	100%
A13	Lido Cerano	Adr	Coastal	*P. oceanica*, sandy, rocky	5400 m^2^	2	0 L; 1 M; 1 S	0.04	100%
A14	Frigole	Adr	Coastal	*P. oceanica*, sandy, rocky	3600 m^2^	1	0 L; 0 M; 1 S	0.03	100%
A15	Torre Specchia	Adr	Coastal	*P. oceanica*, sandy, rocky	3600 m^2^	2	0 L; 2 M; 0 S	0.06	100%
A16	Alimini	Adr	Coastal	*P. oceanica*, sandy, rocky	3400 m^2^	14	6 L; 5 M; 3 S	0.41	100%
A17	Porto Miggiano	Adr	Coastal	*P. oceanica*, sandy, rocky	3600 m^2^	8	4 L; 3 M; 1 S	0.22	100%
L1	Varano Lagoon	Adr	Lagoon	*C. nodosa*, *Zostera* sp., sandy, muddy	6000 m^2^	0	0 L; 0 M; 0 S	0	n.a.
L2	Alimini Lagoon	Adr	Lagoon	*C. nodosa*, *Zostera* sp., sandy, mudy	1800 m^2^	0	0 L; 0 M; 0 S	0	n.a.
I1	Lido Azzurro	Ion	Coastal	*P. oceanica*, *C. nodosa*, sandy	1800 m^2^	2	0 L; 0 M; 2 S	0.11	100%
I2	San Pietro Island	Ion	Islands	*P. oceanica*, *C. nodosa*, sandy, rocky	1800 m^2^	7	2 L; 0 M; 5 S	0.39	100%
I3	San Vito	Ion	Coastal	*P. oceanica*, *C. nodosa*, sandy, rocky	1800 m^2^	3	0 L; 2 M; 1 S	0.17	100%
I4	Torre Ovo	Ion	Coastal	*P. oceanica*, sandy, rocky	1800 m^2^	3	1 L; 2 M; 0 S	0.17	100%
I5	Torre Colimena	Ion	Coastal	*P. oceanica*, sandy, rocky	3600 m^2^	10	3 L; 6 M; 1 S	0.28	100%
I6	Torre Lapillo (MPA)	Ion	Coastal	*P. oceanica*, sandy, rocky	3600 m^2^	18	10 L; 8 M; 0 S	0.50	100%
I7	Porto Cesareo (MPA)	Ion	Coastal	*P. oceanica*, sandy, rocky	4100 m^2^	33	13 L; 14 M; 6 S	0.80	100%
I8	Sant’Isidoro (MPA)	Ion	Coastal	*P. oceanica*, sandy, rocky	1800 m^2^	28	21 L; 7 M; 0 S	1.56	100%
I9	Santa Caterina	Ion	Coastal	*P. oceanica*, sandy, rocky	3400 m^2^	16	7 L; 8 M; 1 S	0.47	100%
I10	Baia verde	Ion	Coastal	*P. oceanica*, sandy, rocky	3600 m^2^	142	41 L; 71 M; 30 S	3.94	100%
I11	Torre San Giovanni	Ion	Coastal	*P. oceanica*, sandy, rocky	3000 m^2^	7	0 L; 4 M; 3 S	0.23	100%
**ADRIATIC SEA**			Coastal & Island		52,500 m^2^	46	13 L; 18 M; 15 S	0.09	100%
**IONIAN SEA**			Coastal & Island		30,300 m^2^	269	98 L; 122 M; 49 S	0.88	100%

*n.a*. not available, *Adr* Adriatic Sea, *Ion* Ionian Sea.

**Fig. 1:**
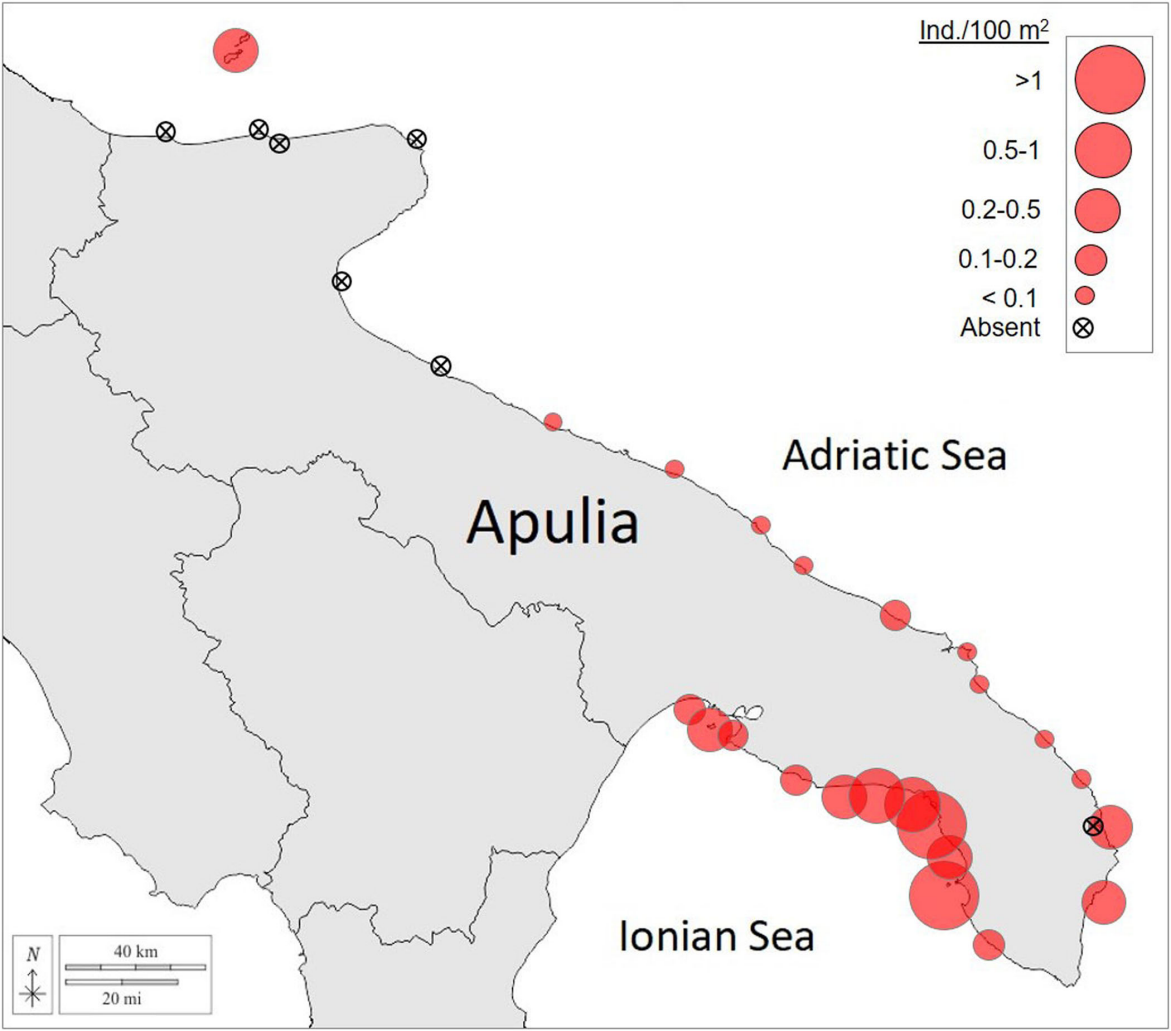
Distribution and local densities of *P. nobilis*. The crossed circles indicate the absence of speciemens, the red circles indicate the presence of dead specimens and their size inicates the local density in terms of dead shell/100 m^2^.

The presence of *P. nobilis* shells was registered in all the 11 monitored sites of the Ionian Coast. In the Adriatic Coast, *P. nobilis* shells were found in the Tremiti Islands (A1) and among the southern sites from A7 to A17. No traces of *P. nobilis* were found in the northernmost sites of the Apulian coast (A2–A6) (Fig. 1).

The density of dead pen shells recorded for each coastal area, reported in Table 1 as the number of individuals/100 m^2^ (Table 1) allowed to estimate the pre-MME density of *P. nobilis*. Substantial differences between the two basins were observed, with the Ionian Sea showing considerably higher densities than the Adriatic Sea, with 0.88 ± 0.34 (mean ± SE) individuals/100 and 0.09 ± 0.03 (mean ± SE) individuals/100 m^2^ respectively. The values recorded in the Ionian Sea were >0.1 individuals/100 m^2^ in all sites. The highest mean value was recorded in I10, located in the Gulf of Gallipoli, with a density of 3.94 ± 0.33 (mean ± SE) individuals/100 m^2^, followed by I6, I7, and I8 sites, where densities of 0.50 ± 0.04 (mean ± SE); 0.80 ± 0.09 (mean ± SE) and 1.56 ± 0.6 (mean ± SE) individuals/100 m^2^ were recorded, respectively.

In the Adriatic basin, the different pen shell densities might indicate an irregularly increasing trend, from North (absence of specimens), to the South, where density of 0.22 ± 0.13 (mean ± SE) (A17) and 0.41 ± 0.10 (mean ± SE) (A16) ind/100 m^2^ have been recorded. Intermediate areas showed densities always lower than 0.1 individuals/100 m^2^ (Fig. 1).

**Fig. 2:**
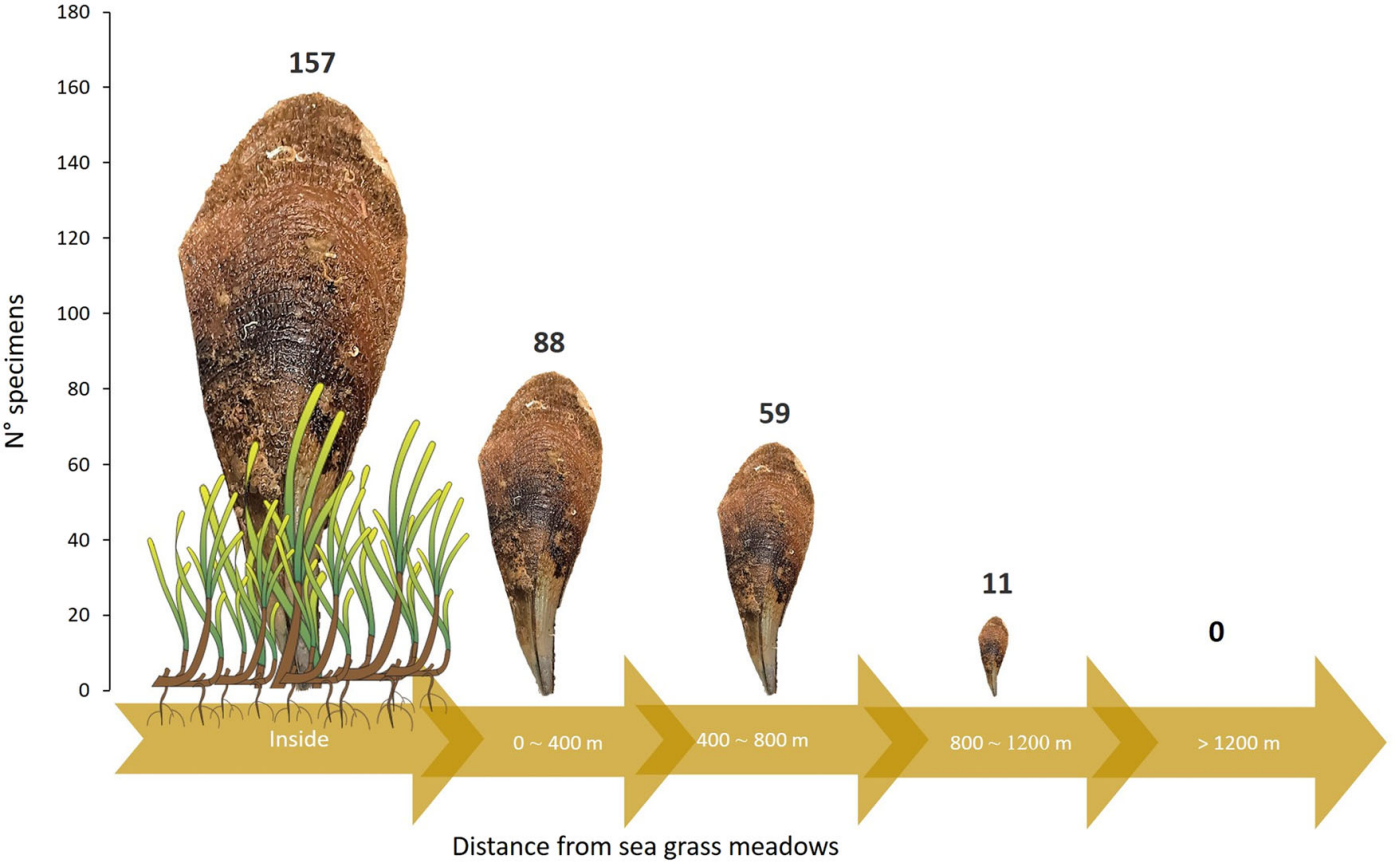
Number of *P. nobilis* specimens detected at four distances from seagrass. The height of icons represents the amount of specimens found at each distance.

Concerning the *P. nobilis* association with seagrass, we note that the highest number of specimens (157), was found inside the meadows. The number of records, moreover, decreased according to the distance from the meadow formations, counting 88, 59, and 11 specimens, respectively, near (0–400 m), away (400–800 m) and far away (800–1200 m) from the meadows. No specimens were found in transects further away than 1200 m from seagrass beds (Fig. 2). The number of specimens, the density values, and the total area investigated for each distance from the seagrass meadows are reported in Table 2.

**Table 2:** Number of *P. nobilis* specimens, areas investigated, and densities in relation to the distance from seagrass meadow boundaries.

Distance from seagrass boundaries	No. of specimens	Investigated area m^2^	Densities ind/100 m^2^
Inside	157	13,380	1.17
0–400	88	13,850	0.64
400–800	59	15,800	0.37
800–1200	11	24,290	0.05
>1200	0	15,480	0

At last, five living *Pinna rudis* adults were also observed during this study, four in I11 and one in I10. All these specimens were observed within the 5 m deep transects.

## Discussion

According to the target of the present study, the mortality incidence on *P. nobilis* in local populations along the Apulia peninsula (the Southeast coast of Italy) following the MME was assessed. In addition, an investigation on the species distribution and densities in the Adriatic and the Ionian Sea was carried out, which allowed us to build a picture of species populations before the MME.

Concerning the *P. nobilis* distribution in the Apulia region before the MME, unfortunately, there is a lack of information at the wide scale, and literature reports only concern semi-enclosed systems such as the Taranto basins^17–19^ and the Aquatina lagoon^20^. No large-scale monitoring program on *P. nobilis*, in fact, has been carried out previously along the Apulian coast, although this kind of surveys is indispensable for the management of a protected species and must become mandatory for a critically endangered species such has become *P. nobilis*. The present data-gathering, that is aimed to partially address this information gap, based on the monitoring of recently dead specimens, allowed to realize a plausible map of *P. nobilis* distribution and densities before the MME in 30 areas distributed along the entire Apulian region coast.

Along the Ionian coast, recently dead *P. nobilis* were detected in all the areas studied, highlighting a continuous distribution of the species prior to the MME, differently from the not continuous distribution along the Adriatic coast. The occurrence of *P. nobilis* was recorded in the areas surveyed in the south, from A7 to A17, but no traces were found along the northernmost areas except for the Tremiti archipelago, suggesting that the northernmost Adriatic coast of the region does not meet the environmental conditions suitable for hosting this species. Nevertheless, in the Gulf of Manfredonia multiple reports from fisherman indicating the presence of the species in a local *Cymodocea nodosa* meadow before the 1980s, suggest that this area may have been an exception in the past. Therefore, we can assume that excessive fishing and anthropogenic activities in this area are likely to have caused the species to disappear many decades ago.

Data regarding the mortality incidence after the MME in Apulian populations is scarce. Panarese et al.^11^ reported the advent of the disease in Mar Piccolo di Taranto but without describing the disease incidence. In this study, a mortality incidence of 100% in all basins, bathymetric (down to 15 m) and habitat types, was recorded, demonstrating the severity of the situation along the entire Apulian coast, both inshore and offshore, and in lagoon and marine-protected areas.

Although the availability of nutrients and the trophic conditions are assumed to be very different between offshore, inshore, and transitional systems, the archipelago of Tremiti islands, located 13 miles away from the coast, showed no differences in mortality incidence from sites along the coast, evidencing the same critical conditions in all environments.

Many Mediterranean lagoon systems, including the Ebro Delta, Mar Menor Lagoon in Spain^21^, the Rhone delta, Leucate and Thau in France^22–24^, Venice, Grado-Marano and Faro in Italy^25–27^, Bizerte in Tunisia^24^ are considered the last healthy shelters for *P. nobilis* populations in the Mediterranean Sea^22^. These systems seem to offer a degree of resistance against the disease and are all characterized by high seasonal fluctuations of environmental parameters, such as temperature and salinity. It has been supposed that the effect of these fluctuations could make these environments less suitable for the spread of the disease and reduce the rate of transmission^21,22^. In the present study, two lagoon systems were also investigated, but no live specimens were found. These systems are strongly affected by the saltwater intrusion and the freshwater inputs became very low during the dry season. Hence, we can assume that during the summer season, when *P. nobilis* become susceptible to the disease, no salinity barrier against the pathogen spread persists in these lagoons systems.

Considering that the lagoon refuges currently represent the main source of larval production for *P. nobilis* recruitment^22,28^, the collapse of these populations confirms the severity of the situation for species conservation. For the Italian coast, the last live populations are those in the lagoons located in the northen Adriatic Sea (Venice and Grado-Marano lagoon). These environments can act as larval exporters for the Adriatic Sea taking advantage of the mobility of the larvae that can spread over hundreds of kms^28^.

Regarding the timeframe of the spread of the MME along the Apulian coast, the first report of the infection dates back to 2018^18^, in the Mar Piccolo di Taranto. Compared to the first MME event observed in the Spanish coast in 2016^5,7^, the disease has spread from the western to the eastern basin of the Mediterranean Sea over a period of 2 years. Our surveys, carried out in 2020, showed that 91% of the shells were still undamaged and with joined valves. Based on the state of conservation of the shells^29^ it is possible to hypothesize that the death of the specimens was a recent phenomenon that had occurred in Apulia in the two years preceding our surveys, and most probably it should be dated back to 2019.

Kersting and Ballesteros^30^ have suggested that other species, such as *P. rudis*, could benefit from the collapse of the *P. nobilis* population. During our surveys, only 5 specimens of *P. rudis* were found, located in 2 sites, but it must be considered that the survey was carried out only a short time after the MME of *P. nobilis*. Further studies aimed at assessing an increase in *P. rudis* in the investigated areas would be of great interest to corroborate this hypothesis.

In these surveys, *P. nobilis* showed transverse distribution among habitat types occurring both in marine and lagoon systems, inside and outside seagrass meadows, on sandy, rocky, and maerl beds substrate. Nevertheless, on a spatial macro (from a few kilometers to tens of kilometers) and mesoscale (from hundreds to thousands of meters), an overlap with the distributional range of seagrass meadows emerges. A clear cross-boundary subsidy trend was evidenced by the data collected on *P. nobilis* distribution in association with seagrasses. The specimens inside seagrass meadows were almost double than those detected nearby and a gradual decrease was observed with the increase of the distance from the seagrass patches (Fig. 2). This is particularly evident along the northern Adriatic coast of the region, where extended seagrass meadows are absent and, no trace of *P. nobilis* was encountered, except in the Tremiti archipelago where both *P. oceanica* meadows and pen shells were found. By contrast, present data reporting *P. nobilis* as associated with various seagrass species, such as *P. oceanica*, *C. nodosa*, and *Zostera* sp., are consistent with the macroscale and mesoscale association between *P. nobilis* and seagrass meadows *sensu lato* and most literature reporting ubiquitous distribution of *P. nobilis* both in lagoon-estuarine^21,22,24–26,31^ and in marine ecosystems^4,7,9,14,16,24^.

However, regarding their microscale distribution, the pen shells in our surveys were recorded also outside the seagrass meadows boundaries, at times up to 1 km away. Hence, seagrass sheltering can potentially be ruled out as the sole explanatory factor for the distribution pattern of the species. The pattern emerging from this study led us to hypothesize that a trophic link with the seagrass detritus food-chain may explain both the macroscale–mesoscale association with seagrass species and the microscale cross-boundary distribution. In fact, seagrass detritus is highly refractory, since it is largely exported to the nearby areas where it can represent the major food source for other invertebrates^32–34^. This hypothesis is consistent with the stomach contents observations reported by Davenport et al.^3^ indicating detritus as the bulk component, accounting for 95% of the total ingested material.

One of the main factors underlying the distribution pattern in benthic invertebrates is indeed food availability^35,36^. According to the Ideal Free Distribution (IFD) theory, the individuals in a population disperse to different resource patches within their environment, minimizing competition and maximizing fitness^37^. When the IFD assumptions are met, the number of individuals who aggregate in patches is proportional to the amount of food resource available in each one. Accordingly, the distribution of large, long life, and sessile organisms such as *P. nobilis* would be expected to depict the species trophic supply, by analyzing the resources available in those patches.

Studies on the seagrass system energy flow have shown that seagrass debris must be fractionated before entering the food chain^33^. In this way, plant material becomes fine particulates moving in the boundary layer over the sediment–water interface^38,39^. These processes take time, and while the matter is transported, heterotrophic bacteria grow exponentially, turning it into a high quality and protein-enriched food for consumers. Hence, bacteria adhering to seagrass detritus may play a key role in this benthic food chain and sediment–water interface consumers may incorporate more energy from associated microbes than from the detritus itself^32,38^. On the basis of these considerations, it is reasonable to hypothesize that the quantity, composition and origin of the suspended particles are regulated by a drift mechanism and that this mechanism may explain local densities of *P. nobilis* as a response to sinking rates and resuspension effects. This hypothesis explain also the species distribution in systems, characterized by strong dominant current and shallow seabeds where the seagrass detritus can be spread/drift several kilometers away from the meadows. An example of this condition is encountered in the north Adriatic Sea (e.g., Gulf of Trieste) where extensive population of *P. nobilis* develops on several sink areas even kilometers downstream from the meadows. The assumption of the species’ ability to feed on seagrass detritus, together with the high biomasses reached (large size specimens and high density), lead us to suppose that *P. nobilis* may play a key role in the processing of matter and in the energy pathway deriving from seagrass detritus in Mediterranean coastal areas. This makes the repercussions of the MME not only a problem of conservation, but also and above all, an ecological-functional issue.

We can, therefore, conclude that Mediterranean seagrass meadows not only constitute a habitat for *P. nobilis*, but probably also a food source through refractory detritus generation which is transferred and transformed outside the meadows. Unfortunately, literature is lacking on this topic and further investigations are needed to define the trophic role and function of these filter feeders in the different seagrass meadows.

The density values that emerged were significantly different among basins. In the Adriatic Sea, where all the coastal values were recorded, the densities were consistently lower than those reported in the Ionian Sea, except for the two southernmost areas. In the Adriatic basin, it was also possible to recognize a north-south trend when considering the densities of pen shells in the coastal areas. Although the values recorded along the southern coast of the region were much greater than those recorded in the central coast, they were far lower than those reported by Čižmek et al.^40^ in the Croatian coast (North Adriatic Sea). Similar values to ours within the same basin were reported by Celebicic et al.^41^ in Bosnian waters (0.12 individuals/100 m^2^).

On the other hand, in the Ionian areas, the values recorded were consistently >0.1 individuals/100 m^2^. The values recorded in the Mar Grande di Taranto were higher than those reported by Centoducati et al.^17^ (0.1–0.7 ind/ha^2^). From interviews with fishermen, it emerged that illegal trawling in this area has strongly impacted the natural populations of the Mar Grande di Taranto, and a partial reduction of this activity, in recent years could explain the slight increase in density compared to the 2004 survey data^17^.

In interpreting our data, it should be considered that the surveys were carried out employing an extensive sampling protocol conceived to assess wide surface densities on coastal areas investigating across several habitat types. Therefore, literature density values focused only on local areas or habitat patchiness that were not randomly selected must be contextualized when compared with these data. In addition, given the scale of the presented surveys, emphasis must be given to *P. nobilis* absence data of which the literature appears poor. Indeed, contrary to the data on presence, reliable absence data are difficult to obtain requiring much greater effort to rule out a rare occurrence^42^. The absence data obtained in this study derive from the merger of two different data types. The first come from the local ecological knowledge obtained from interviews with the local fishermen, which allowed us to confirm our data, excluding spot occurrences in the same areas. Furthermore the interviews allowed us to collect information on a historical series of species presence/absence in the areas, which was helpful to confirm local absence when no *P. nobilis* specimens were recorded in our surveys. The second derives from the complete vision of divers during the field surveys. Indeed the scuba diver’s view was at least 10 times wider than 50 cm from the side around the rope and hence, the perception of absence can be extended over a much larger surface area investigated. By merging these two sources of information, we can assume that the absence data collected in exhaustive and complete.

In conclusion, this study investigated different basins, habitat types, and bathymetries along the Apulian coast. The shells spatial distribution that arise from this study allowed to obtain important information on the species trophic ecology. Indeed, the species distributional pattern showed a strong overlap with seagrass meadows on meso and macro geographical scale, however this was not the case on a micro scale. This result indicates that although there is a strong relationship between *P. nobilis* and seagrass meadows, it is not limited to the habitat patch but crosses the boundaries of seagrass. This result led us to hypothesize that the distribution of *P. nobilis* displays a trophic link through the cross-boundary subsidy occurring from seagrass meadows to the nearby habitat, by means of the refractory detrital pathway. However, further investigations taking into account other factors such as hydrodynamics, are needed to investigate this topic.

No live specimens of *P. nobilis* were found in >800 km of coastal line, leading us to the conclusion that the coastal and lagoon population had totally collapsed in the region after the MME. The seriousness of the situation on the Apulian coasts, just as in the other Mediterranean ecoregions, indicates that the MME that began in 2016 is still in progress, and no local population can be considered safe. Given the gravity of the current situation, it is vital for species preservation to extend the survey across the entire Italian coast to gain a overall picture of the status of the *P. nobilis* population on a national scale. Indeed, other regions may reveal the existence of natural shelters, where live populations of *P. nobilis* may still persist. If this is the case, it is essential to identify and protect them in time. As already suggested by Kersting et al.^9^, this initiative should be conducted in parallel by all the nations of the Mediterranean basin to implement standard guidelines for the monitoring, protection, and recovery of this critically endangered species.

## Methods

**Fig. 3:**
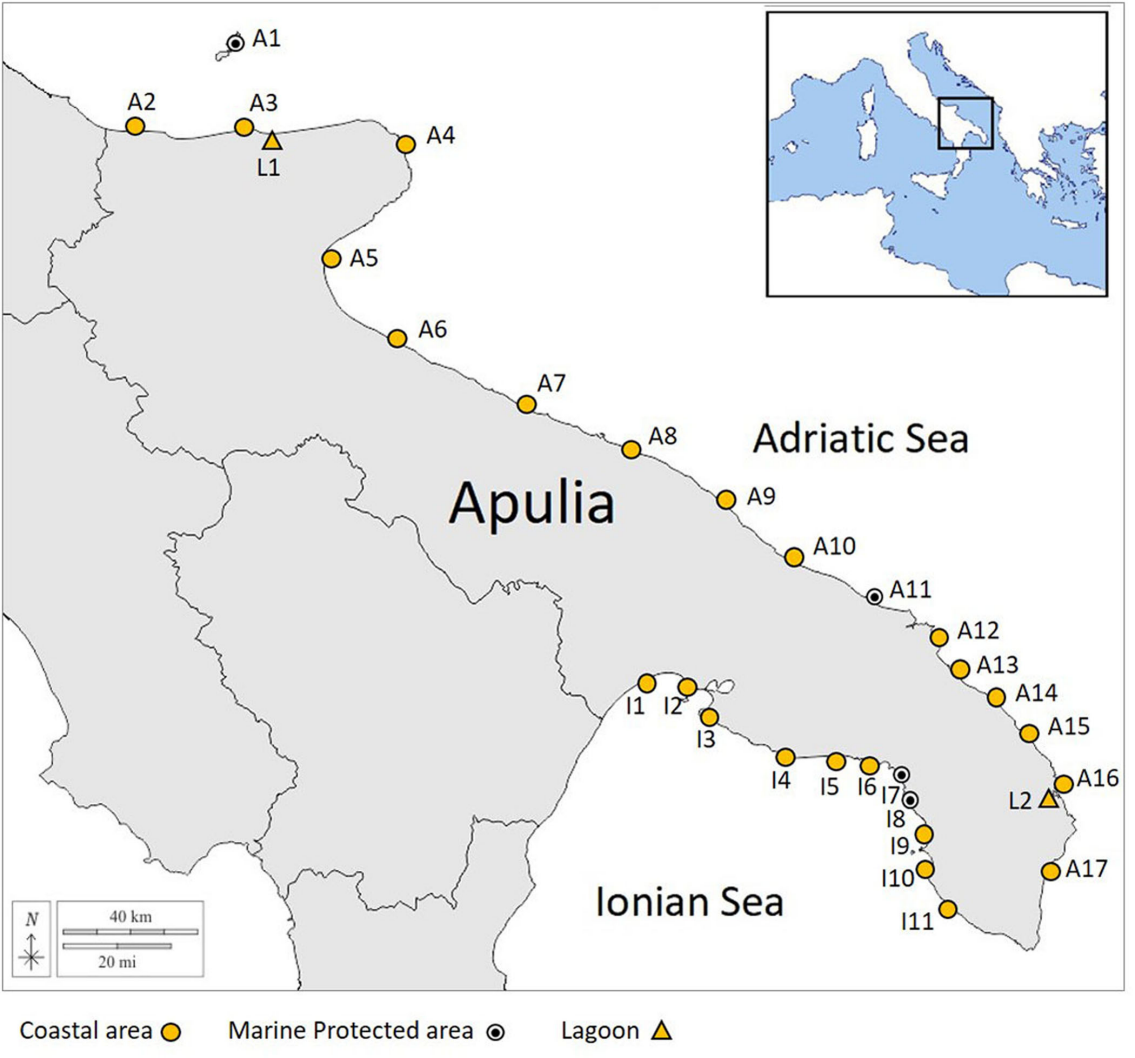
Surveyed areas investigated along the Apulian coast. Yellow circles indicate coastal Areas, black dotted circles indicate marine protected areas and yellow triangles indicate lagoon systems.

**Fig. 4:**
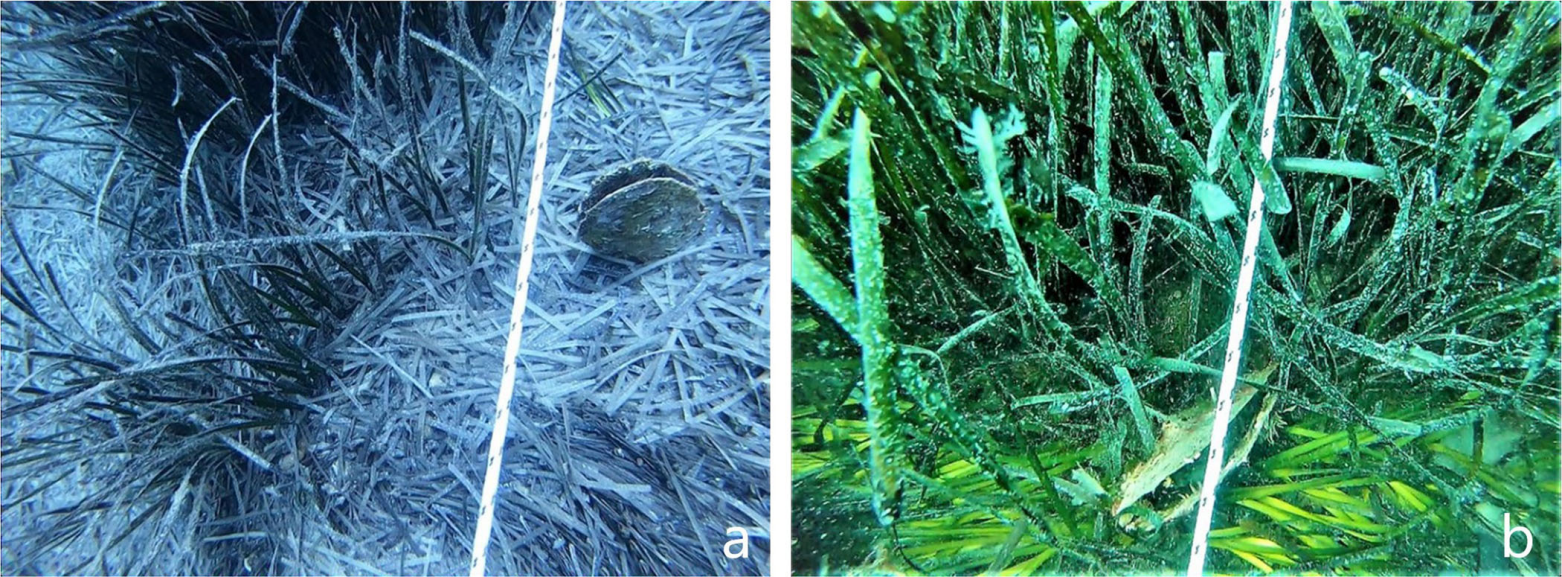
Monitoring of *P. nobilis* during the field surveys. Underwater images show the shells beside the rope at a depth of 15 m (**a**) and 5 m (**b**).

To provide data on the *P. nobilis* distribution and impact of the MME at a subregional scale, field surveys were conducted in the summer 2020 by specially trained scuba divers. Thirty areas were investigated along almost 800 km of Apulia coast, 19 of which located in the Adriatic Sea and 11 in the Ionian Sea.including the three marine protected areas of the region (Torre Guaceto, Porto Cesareo and the Tremiti archipelago) and two transitional systems (Varano and Alimini) (Fig. 3). Three bathymetric levels were examined (5, 10, and 15 m), employing the Navionics SonarChart to draw the transects, based on the sonar cartographic bathymetry. the extensive transects thus conducted, through a cross-boundary approach allowed to cover almost all the habitat types shallower than 15 m depth, as it would have been too much onerous and logistically complicated to extended the investigation deeper. The actual depth of the transects was checked through the vessel echo sounder to adjust the path, if necessary, with field data. For each bathymetric level, one or more transects were defined by laying down a 600 m-long line (labeled every 10 m with popups) stretched out parallel to the coastline. The line was held down by anchors positioned every 100 m, and the GPS tracking was collected during the positioning. Two marker buoys were placed at the endpoints of each transect to signal their location at the surface. For each survey, the scuba divers covered a linear transect from 400 to 600 m long (depending on the diving time) by counting and recording specimens within a 1 m span (50 cm from the line on each side), for a total survey area of 400–600 m^2^ for each transect (Fig. 4). When the transect passed through seagrass meadows, the diver moved aside the seagrass leaves around the rope manually to detect all the *P. nobilis* specimens. From preliminary surveys, 50 cm to 1 m was found to be the optimal width for a scuba diver to investigate the macrobenthic organisms thoroughly when separating the seagrass meadows leaves. Each scuba diver was equipped with high-resolution head-camera in order to record videos for each transect (Fig. 4 shows snapshots from the records), and a ruler in order to measure the size of the pen shells encountered. The total length of the pen shell (TL) specimens was calculated by summing the unburied height to the estimated buried length (BL), this last one was derived from the shell width at the substrate level (Sw), following the equation, BL = 1.79 × Sw^43^.

**Fig. 5:**
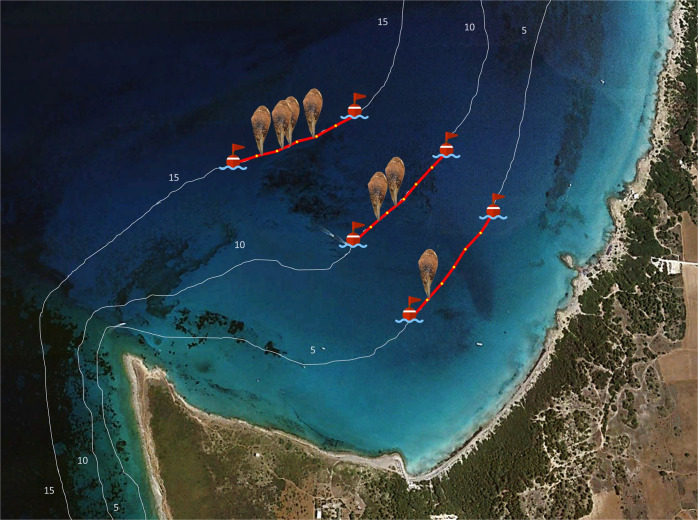
The image shows the spatial distribution of *P. nobilis* specimens in a study area. The *P. nobilis* icones represent the map-matching projections of specimen positions along GPS tracers of the survey transects (three out of six transects in Baia Verde, Gallipoli 5, 10, and 15 m).

The videos recorded by the head cameras during each surveyed transect were analyzed in order to obtain data on the pen shell position as well as biometric and habitat information. From each transect, data on specimen position were recorded progressively every 10 m, following the numerated popup labels along the 600 m long transect line. These progressive data allowed us to define the position of each *P. nobilis* specimen along the georeferenced transect. The position of each individual was projected onto the GPS tracking of each transect, obtaining the spatial distribution of the specimens within the areas surveyed (Fig. 5). Using field habitat data records and the specimen’s projections both on seagrass chart (EuSeaMap) and satellite images through Google Earth Engine, the approximate distance of the specimens to the nearest seagrass meadows was defined. Based on these data, each specimen was assigned to the following four groups: inside the meadows, near the meadows (from 0 to 400 m away), away from the meadows (from 400 to 800 m), and far away from the meadows (from 800 to 1200 m).

During the surveys, specimens that had clearly been dead for a long time (based on the shell state and fouling inside the shell) were not considered (Katsanevakis et al.^44^). After each survey, line, ballast, and buoys were winched on board. A total of 161 transects were completed, for an overall survey length of 90 km.

At each location, 4–12 fishermen were individually interviewed in order to collect historical and up-to-date information on *P. nobilis* densities, distribution, size classes, interaction with human activities (mostly fishing), local habitat association, and effects of storm events and mortality occurrence.

## Data Availability

The data are available on request from the corresponding author, A.R.
